# Impact of fish consumption on disability progression in multiple sclerosis

**DOI:** 10.1136/jnnp-2024-335200

**Published:** 2025-02-25

**Authors:** Eva Johansson, Jie Guo, Jing Wu, Tomas Olsson, Lars Alfredsson, Anna Karin Hedström

**Affiliations:** 1 Department of Clinical Neuroscience, Karolinska Institutet, Stockholm, Sweden; 2 Department of Nutrition and Health, China Agricultural University, Beijing, Beijing, China; 3 Karolinska Institute, Stockholm, Sweden

**Keywords:** MULTIPLE SCLEROSIS, Patient Outcome Assessment, EVIDENCE-BASED NEUROLOGY

## Abstract

**Background:**

Emerging evidence indicates that diet, including fish consumption, may play a role in the development and progression of multiple sclerosis (MS). We aimed to investigate the influence of fish consumption on disability progression in MS.

**Methods:**

Incident cases from the population-based case-control study Epidemiological Investigation of MS (n=2719), with data on fish intake and Expanded Disability Status Scale (EDSS) outcomes, were categorised by fish consumption and followed up to 15 years post-diagnosis through the Swedish MS registry. A digital follow-up questionnaire in 2021 assessed changes in fish intake over time (n=1719). Cox regression models, adjusted for clinical and demographic variables, were used to evaluate 24-week confirmed disability worsening (CDW), and progression to EDSS 3 and EDSS 4.

**Results:**

Higher total consumption of lean and oily fish at diagnosis was associated with a reduced risk of CDW (HR 0.66, 95% CI 0.51 to 0.86), EDSS 3 (HR 0.55, 95% CI 0.39 to 0.79) and EDSS 4 (HR 0.57, 95% CI 0.33 to 0.96) compared with low consumption. These associations showed significant trends and remained consistent after further adjustment for various lifestyle factors. The protective effects were more pronounced among patients who maintained consistent fish consumption during the follow-up period.

**Conclusions:**

Our findings suggest that higher fish consumption is associated with more favourable MS disability progression, supporting diet as a potentially modifiable factor. Replication and validation are needed before transfer to practice.

**WHAT IS ALREADY KNOWN ON THIS TOPIC**

- While cross-sectional studies have linked higher fish consumption to reduced multiple sclerosis MS) severity, there is limited longitudinal evidence on the impact of fish consumption on MS disability progression.

**WHAT THIS STUDY ADDS**

- This study provides robust longitudinal evidence that higher total fish consumption (lean and oily fish combined) is associated with a reduced risk of MS disability progression, including confirmed disability worsening (CDW) and progression to Expanded Disability Status Scale (EDSS) 3 and EDSS 4.

**HOW THIS STUDY MIGHT AFFECT RESEARCH, PRACTICE OR POLICY**

- The results underscore the potential role of diet, particularly fish consumption, as a modifiable factor that could complement existing therapeutic strategies for MS. They highlight the need for further research to validate these findings and investigate the underlying biological mechanisms.

## Background

 Multiple sclerosis (MS) is a complex inflammatory and neurodegenerative disorder of the central nervous system, and its occurrence is correlated with several genetic and environmental factors.^1^ Evidence suggests that diet may play a role in the development of inflammatory diseases, including MS.^2 3^ An association between fish consumption and the risk of developing MS has been observed,^4^,^6^ and cross-sectional surveys have suggested that higher fish consumption is associated with more favourable disability outcomes in MS. For example, a study in Italy found that a Mediterranean diet, including regular fish consumption, correlated with lower disability status in MS patients.^7^ Another study linked fish intake and omega-3 supplementation to lower disability in MS.^8^ Additionally, a European study observed a modest association between higher fish intake and slower MS progression.^9^ However, there is a lack of prospective studies examining the relationship between fish consumption and the progression of MS-related disability.

Despite advancements in high-efficacy disease-modifying therapies, which have significantly reduced relapse rates and shown benefits in slowing progression for some patients, preventing disease progression remains a major challenge. Understanding the impact of environmental exposures and lifestyle habits on disease progression is increasingly important for optimising patient outcomes. To address this, we followed patients with MS from an incident population-based case-control study to examine the influence of fish consumption habits on MS disease progression. Additionally, we investigated the effect of changes in fish consumption after diagnosis on disability progression.

## Methods

The Epidemiologic Investigation of Multiple Sclerosis (EIMS) is a Swedish nationwide population-based case-control study. Between April 2005 and June 2015, EIMS recruited 2880 newly diagnosed MS patients from hospital-based neurology units and privately run clinics across Sweden. The response rate among cases was 93%. All patients were diagnosed according to the McDonald criteria.^10 11^ All participants provided information on environmental exposures and lifestyle habits at study inclusion, by completing a standardised questionnaire. They were also asked to provide blood samples for genetic and serologic analyses. More details on the study design and methods are provided elsewhere.^12^ Of the 2880 patients, 2767 (96%) were followed-up with Expanded Disability Status Scale (EDSS) scores in the Swedish MS registry. Patients with incomplete information regarding fish consumption habits were excluded (n=48). The present study thus comprised 2719 patients with MS.

To explore the influence of lifestyle changes post-diagnosis, participants were asked to complete a digital follow-up questionnaire in 2021, capturing lifestyle habits from the time of diagnosis. Of the 1821 patients who completed the questionnaire (response rate 67%), 1719 were followed up with EDSS in the Swedish MS registry. The study was approved by the regional ethical review board at Karolinska Institute and was conducted in accordance with the ethical standards of the 1964 Declaration of Helsinki and its later amendments.

### Definition of exposure

Participants were asked separately and specifically about their average consumption of lean and oily fish, as these questions were not part of a full food frequency questionnaire due to the already extensive baseline questionnaire. Oily fish species were defined as species with a fat content >3%, such as herring, mackerel, tuna, salmon, and trout. Lean fish were defined as species with a fat content <3%, such as cod, pollock, haddock, whiting, and pike perch. Responses were recorded on a 4-point scale: never/seldom, 1–3 times/month, weekly, or daily. Due to the very few participants reporting daily consumption fish consumption, the last two categories were merged into one. We additionally constructed a frequency score for fish consumption by summing the responses, yielding a value between 2 (lowest exposure) and 6 (highest exposure). The fish consumption frequency score is illustrated in table 1.

**Table 1:** Fish consumption habits at baseline

	Never or seldom oily fish		Oily fish 1–3 times/month		Oily fish weekly	
Never or seldom lean fish	FS=2	332 (12)	FS=3	392 (14)	FS=4	94 (3.5)
Lean fish 1–3 times/month	FS=3	193 (7.1)	FS=4	1012 (37)	FS=5	268 (9.9)
Lean fish weekly	FS=4	36 (1.3)	FS=5	168 (6.2)	FS=6	224 (8.2)

FS, frequency score.

### Measurement of vitamin D (ng/mL)

For patients recruited between 2005 and 2009, vitamin D status was measured as levels of 25-hydroxy-vitamin D using a chemiluminescent immunoassay from Diasorin (Diasorin AB, Sundbyberg, Sweden) and a LIASON instrument provided by Diasorin AB with equimolar measurement of both 25-hydroxy-vitamin D_2_ and D_3_.

### Outcome measures

The Swedish MS registry is used in all neurology units across the country and is integrated into the clinical documentation system.^13^ For each patient, data are continuously recorded by healthcare professionals regarding medical treatment, disease activity, and physical functioning.

To study changes in disability over time, the baseline was defined as the date of the first recorded EDSS. Confirmed disability worsening (CDW) was defined as an increase in the EDSS score by at least 1 point from baseline, sustained between two follow-up visits separated by no less than 6 months (1.5 points if EDSS at baseline was 0, 0.5 points if the baseline EDSS ≥5.5). Time to milestones EDSS 3 and 4 were limited to subgroups of patients with a baseline EDSS <3.

### Statistical analysis

Categorical variables were summarised using frequency and percentage. Continuous variables were summarised using mean and SD. Time to 24-week CDW and the milestones EDSS 3 and 4 endpoints were analysed using multivariable Cox proportional hazard regression. Follow-up time was calculated from baseline until the onset of the events of interest, drop-out, death, or end of follow-up, whichever occurred first. The proportional hazard assumption was tested using Schoenfeld residuals, and no violations of proportionality were observed. P values for trend were calculated using a variable representing the ordered categories of fish consumption habits and each outcome variable.

Due to a moderate correlation between lean and oily fish intake (correlation coefficient 0.4, p<0.0001), it was not feasible to examine high intake of one type while holding the other constant. Therefore, we conducted analyses in two ways: by calculating a fish consumption frequency score to capture overall intake, and by studying specific combinations of lean and oily fish intake to assess the individual contributions of each fish type. To assess the effect of persistent fish consumption habits on MS progression scores, we performed the analysis limited to those who had not changed their fish intake between diagnosis and the time of completing the follow-up questionnaire in 2021. Those who had the same fish consumption frequency score during the follow-up were defined as having persistent fish consumption.

All analyses controlled for age at diagnosis, sex, county (n=21), ancestry (Nordic or non-Nordic), disease phenotype, disease duration, baseline EDSS, and disease-modifying therapy. To account for treatment effects, we calculated the proportion of the duration of follow-up spent on disease-modifying therapy. In a supplementary analysis, we adjusted for additional potential confounders, including physical activity, body mass index (BMI), smoking, snuff use, alcohol consumption, sun exposure, and vitamin D. Physical activity was assessed by asking participants to select one of four levels of leisure-time activity for the past year, which were categorised as follows: predominantly sedentary activities, moderate activity without sweating, regular exercise 1–2 times/week with sweating, and regular exercise ≥3 times/week with sweating. BMI at diagnosis was calculated using self-reported height and weight and categorised into underweight, normal weight, overweight, or obese according to cutoffs used by the WHO. Smoking was dichotomised into current smoking or non-smoking. Alcohol consumption was categorised into no consumption, low, moderate, or high consumption according to cutoffs used by Statistics Sweden.^14^ Snuff use was dichotomised into yes or no. Sun exposure was assessed using three questions regarding ultraviolet radiation exposure. Each answer alternative was reported on a 4-point scale, and by adding the numbers together we constructed an index ranging between 3 (the lowest exposure) and 12 (the highest exposure).^15^ While these variables are theoretically relevant, their inclusion did not substantially alter the primary association between fish consumption and MS progression observed in our main analysis. To minimise the risk of over-adjustment, the final model included age, sex, disease phenotype, disease duration, and treatment.

We conducted several supplementary analyses. A stratified analysis by BMI (using 25 kg/m^2^ as cut-off) was performed to evaluate whether BMI modified the associations between fish consumption and MS outcomes. In a subanalysis (n=1220), vitamin D levels at baseline were adjusted for as a continuous variable alongside sampling month. To assess potential selection bias, baseline characteristics were compared between participants who completed the follow-up study and those who did not, using χ^2^ tests for categorical variables and t-tests for continuous variables. Additionally, to account for potential variations in clinical assessments and practices over the recruitment period, we conducted a sensitivity analysis specifically focusing on the subset of participants enrolled during the peak 5-year period (2007–2011) to evaluate whether the results held in a cohort of patients who were diagnosed within a narrower timeframe. All analyses were conducted in Stata version 17.0 (StataCorp, College Station, TX, USA) and Statistical Analysis System (SAS) version 9.4.

## Results

We included 2719 patients with MS from the initial cohort of 2880, excluding those with incomplete fish consumption data (n=48) and those without EDSS follow-up data (n=63). These patients were followed for up to 15 years. The mean (SD) age at diagnosis of MS and inclusion in EIMS was 38 (11) years. There was no significant difference in baseline EDSS scores by fish consumption habits. Baseline characteristics of cases of the overall sample and by fish consumption habits are presented in table 2.

**Table 2:** Baseline characteristics of overall sample and by fish consumption habits

		Frequency score for fish consumption					P value
	Total	2	3	4	5	6	
N	2719	332	585	1142	436	224	
Age at diagnosis (SD)	38 (11)	35 (10)	36 (11)	37 (11)	40 (12)	40 (11)	<0.0001
Female, n (%)	1950 (72)	233 (70)	420 (72)	814 (71)	321 (74)	162 (72)	0.86
Nordic origin, n (%)	2173 (81)	256 (77)	442 (77)	935 (83)	366 (85)	174 (80)	0.001
Treatment (%)	2573 (95)	322 (97)	563 (96)	1078 (94)	399 (92)	211 (94)	0.004
MS phenotype							0.20
Relapsing, n (%)	2542 (93)	317 (95)	555 (95)	1059 (93)	405 (93)	206 (92)	
Progressive, n (%)	131 (4.8)	10 (3.0)	23 (3.9)	64 (5.6)	19 (4.4)	15 (6.7)	
Unknown, n (%)	46 (1.7)	5 (1.5)	7 (1.2)	19 (1.7)	12 (2.8)	3 (1.3)	
Disease duration, years (SD)	2.6 (3.8)	2.4 (3.4)	2.6 (3.9)	2.7 (3.8)	2.6 (3.7)	2.9 (3.9)	0.46
Baseline EDSS (SD)	1.8 (1.4)	1.8 (1.4)	1.7 (1.4)	1.8 (1.4)	1.8 (1.5)	1.8 (1.5)	0.51
Sun exposure index (SD)	6.2 (1.8)	6.0 (1.9)	6.3 (1.9)	6.1 (1.8)	6.3 (1.8)	6.4 (1.8)	0.002
Vitamin D, ng/mL (SD)^*^	62 (27)	57 (26)	62 (27)	63 (27)	60 (23)	67 (31)	0.08
Past IM, n (SD)	485 (18)	64 (19)	109 (19)	202 (18)	81 (19)	29 (13)	0.18
Physical activity (SD)	2.3 (1.0)	2.1 (1.0)	2.2 (1.0)	2.3 (0.9)	2.5 (0.9)	2.5 (1.0)	<0.0001
Body mass index (SD)	25.1 (4.8)	25.6 (4.9)	25.1 (5.0)	25.1 (4.8)	24.7 (4.6)	24.5 (4.5)	0.03
Current smoking, n (%)	614 (23)	89 (27)	152 (26)	248 (22)	77 (18)	48 (21)	0.007
Alcohol (g/week, SD)	43 (63)	45 (72)	42 (58)	44 (61)	44 (72)	41 (54)	0.37

* data was available for 1220 patients

EDSS, Expanded Disability Status Scale; IM, infectious mononucleosis; MS, multiple sclerosis.

When analysing lean and oily fish separately, weekly intake was associated with a reduced risk of CDW compared with those who seldom consumed fish (HR 0.84, 95% CI 0.71 to 1.00 for lean fish, and HR 0.81, 95% CI 0.68 to 0.97 for oily fish).

There was a moderate correlation between consumption of lean and oily fish (correlation coefficient 0.4, p<0.0001). As a result, it was not feasible to examine the frequency of one type of fish consumption while holding the other constant. However, when participants were grouped based on specific combinations of lean and oily fish frequencies, those with higher intakes of both types of fish experienced the most significant reduction in the risk of CDW (table 3).

**Table 3:** HR with 95% CI of clinical disease worsening post-diagnosis, by frequency of oily and lean fish consumption

Confirmed disability worsening (CDW)						
Oily fish	Lean fish	N	Years (SD)	Outcome (%)	HR (95% CI)*	HR (95% CI)†
**Categorisation 1**						
Seldom	Seldom	332	6.3 (4.3)	173 (52)	1.0 (reference)	1.0 (reference)
Seldom	At least 1–3 times/month	486	6.5 (4.6)	235 (48)	0.90 (0.74 to 1.10)	0.88 (0.72 to 1.08)
At least 1–3 times/month	Seldom	229	6.2 (4.2)	126 (55)	1.04 (0.85 to 1.36)	0.96 (0.76 to 1.21)
At least 1–3 times/month	At least 1–3 times/month	1672	6.6 (4.5)	832 (50)	0.90 (0.70 to 0.98)	0.83 (0.70 to 0.98)
**Categorisation 2**						
Less than weekly	Less than weekly	1929	6.4 (4.5)	975 (51)	1.0 (reference)	1.0 (reference)
Less than weekly	Weekly	362	6.6 (4.4)	185 (51)	1.00 (0.85 to 1.18)	1.00 (0.85 to 1.18)
Weekly	Less than weekly	204	6.4 (4.4)	113 (55)	1.14 (0.93 to 1.40)	1.05 (0.88 to 1.33)
Weekly	Weekly	224	6.9 (4.7)	93 (42)	0.75 (0.60 to 0.95)	0.72 (0.57 to 0.90)

* Crude.

† Adjusted for age at diagnosis, sex, residential area, ancestry, disease phenotype, disease duration, baseline EDSS, and disease-modifying therapy.

EDSS, Expanded Disability Status Scale.

### Fish consumption frequency score (ranging from 2–6)

**Figure 1:**
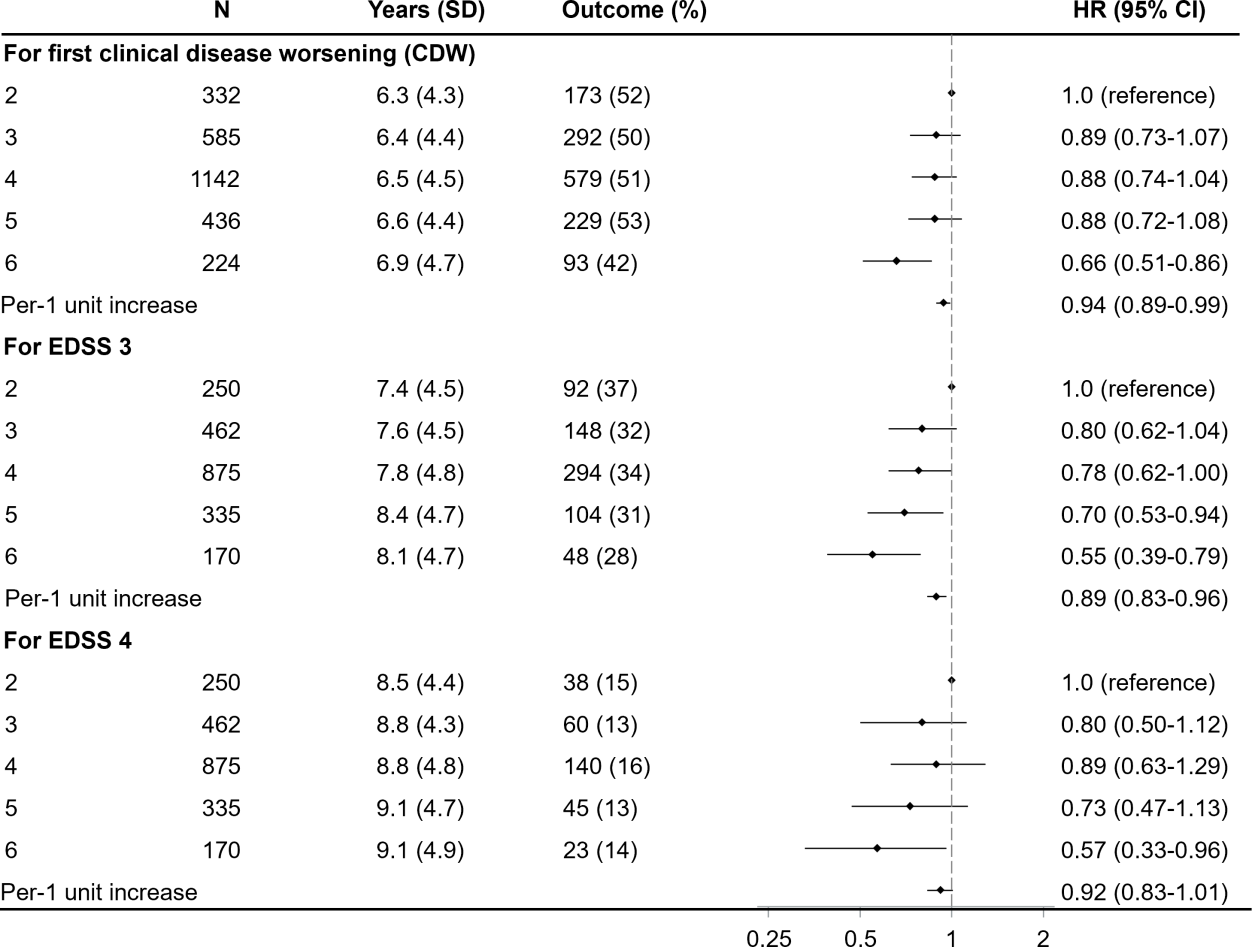
HR with 95% CI of having unfavourable outcomes post-diagnosis, by fish consumption habits *at diagnosis*. Adjustments were made for age at diagnosis, sex, residential area, ancestry, disease phenotype, disease duration, baseline EDSS, and disease-modifying therapy. CDW, confirmed disability worsening; EDSS, Expanded Disability Status Scale.

A fish intake frequency score was developed to assess the overall intake of lean and oily fish, capturing a broader pattern of fish consumption. The highest fish consumption at diagnosis was associated with a reduced risk of CDW (adjusted HR 0.66, 95% CI 0.51 to 0.86), as well as a lower risk of reaching EDSS 3 (adjusted HR 0.55, 95% CI 0.53 to 0.79) and EDSS 4 (adjusted HR 0.57, 95% CI 0.33 to 0.96) compared with the reference group (figure 1, online supplemental table 1). Trends revealed a lower risk of the CDW and progression to EDSS 3 and 4 with increasing fish consumption (figure 1, online supplemental table 1).

### Persistent fish consumption habits during follow-up

**Figure 2:**
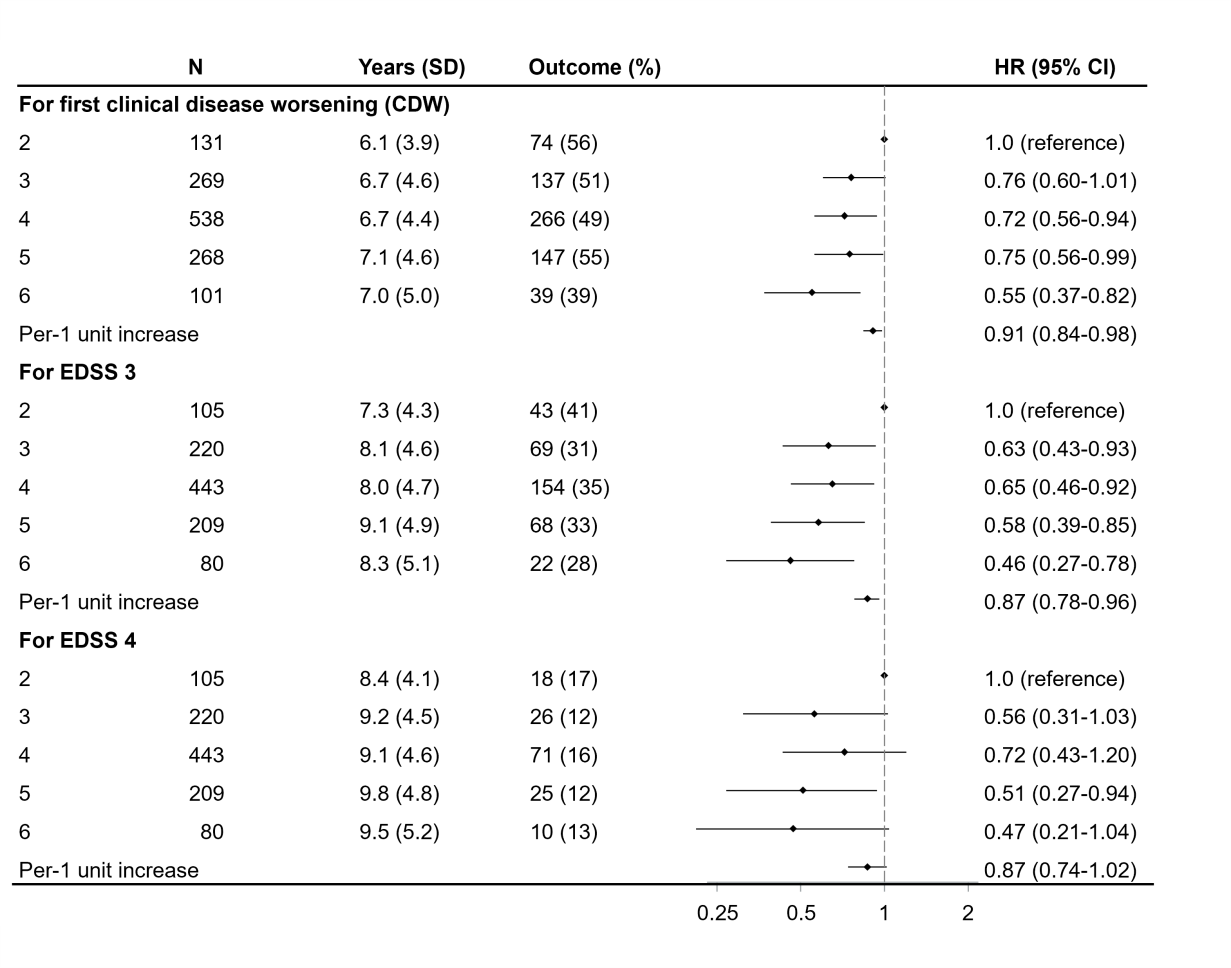
HR with 95% CI of having unfavourable outcomes post-diagnosis, by fish consumption habits at diagnosis. No changes in fish consumption during *follow-up*. Adjustments were made for age at diagnosis, sex, residential area, ancestry, disease phenotype, disease duration, baseline EDSS, and disease-modifying therapy. CDW, confirmed disability worsening; EDSS, Expanded Disability Status Scale.

Of the 1719 patients who completed the follow-up questionnaire in 2021, 412 (24%) had altered their fish consumption frequency (288 increased and 124 decreased their fish intake). The associations between fish consumption and favourable outcomes were more pronounced when the analysis was limited to patients who maintained consistent fish consumption during the follow-up period (figure 2, online supplemental table 4).

### Changed fish consumption habits during follow-up

Those who increased their fish consumption from a frequency score of 2–3 to a score of 5–6 within 5 years post-diagnosis (n=133) had a significantly reduced risk of CDW (HR 0.80, 95% CI 0.63 to 0.99), compared with those who continued to have a low fish consumption (n=400). Although only 16 individuals increased their fish consumption from a baseline score of 2 to a score of 5–6, they exhibited a notably lower HR of CDW of 0.41 (95% CI 0.17 to 0.95), compared with those who remained at the lowest level (n=101).

### Supplementary analyses

Our findings remained similar when physical activity, BMI, smoking, alcohol, and sun exposure were accounted for (online supplemental table 2). They also remained similar when we further adjusted for vitamin D and sampling month (online supplemental table 3). When we stratified the analysis by BMI, the HR of CDW was 0.71 (95% CI 0.51 to 0.98) among those with BMI ≤25, and 0.64 (95% CI 0.43 to 0.94) among those with BMI >25. In the subset of participants recruited between 2007 and 2011, the association between high fish consumption and risk of CDW remained consistent (HR 0.67, 95% CI 0.46 to 0.97), and the trend of decreasing risk of CDW with increasing consumption of fish was maintained (HR 0.93, 95% 0.87 to 1.00).

To address potential selection bias, we compared baseline characteristics between those who responded to the follow-up questionnaire and those who did not. Non-responders were more likely to be of non-Nordic ancestry, smokers, report lower physical activity levels, and be untreated. Otherwise, there were no significant differences between the groups in variables such as age, sex, MS phenotype, disease duration, EDSS, or fish intake, suggesting that the follow-up cohort is broadly representative of the original study population (online supplemental table 5).

## Discussion

Our findings suggest that higher fish consumption is associated with a reduced risk of EDSS-related disability progression in MS, and highlight the potential importance of dietary habits in managing the disease.

Our findings align with cross-sectional surveys indicating that higher fish consumption is linked to more favourable disability outcomes in MS.^7^,^9^ We observed consistent associations across the three disability measures, indicating that higher fish consumption may exert a stable beneficial impact across different stages of MS progression. This internal consistency in the results supports the robustness of our findings and suggests that fish consumption may offer continuous benefits as MS disability progresses.

Our analysis also revealed that patients who consistently consumed high levels of fish over time experienced a more pronounced inverse association. This consistency might be important because the anti-inflammatory and neuroprotective benefits of nutrients found in fish may accumulate over time, leading to sustained improvements in health outcomes.

Interestingly, patients who increased their fish consumption after their diagnosis also showed a reduced risk of CDW. This finding suggests that dietary modifications post-diagnosis can still positively influence disease progression.

Fish is a rich source of several nutrients that might benefit individuals with MS, namely, omega-3 fatty acids and taurine. Omega-3 fatty acids are well-known for their anti-inflammatory properties and have been associated with reduce inflammation associated with MS.^16^ While omega-3 fatty acids, predominantly found in oily fish, may contribute to reduced disability progression, the beneficial effects observed from lean fish consumption suggest that other factors may also play a significant role. One such factor is taurine, an amino acid found in significant amounts in fish and seafood.^17^ Taurine is the most abundant free amino acid in the brain and, although there are endogenous mechanisms for its production, an exogenous supply is necessary to meet physiological needs.^18^ Taurine has diverse cellular functions, including cytoprotective actions through antioxidative and anti-inflammatory effects, making it a potential therapeutic agent for neurological disorders.^17^

Furthermore, diet profoundly influences gut microbiota, which has been shown to influence the production and metabolism of fatty acids, with potential immunomodulatory effects.^3 19^ Therefore, the beneficial impact of fish consumption on MS progression might also be mediated through favourable modifications of gut microbiota composition and function.

Despite a weak correlation between oily fish consumption and vitamin D levels (correlation coefficient 0.09, p=0.003), our analysis showed that vitamin D only marginally influenced the association between oily fish consumption and disability progression. Additionally, there was no trend between baseline vitamin D levels and disability progression (HR 1.00, 95% CI 0.996 to 1.002), suggesting that vitamin D may not be the primary factor driving the observed benefits of fish consumption on disability progression in MS.

The strengths of our study are the population-based design using incident cases of MS defined by established criteria, the high response rate, and the detailed information regarding a variety of exposures which makes it possible to consider several potential confounding factors. The patients were followed for up to 15 years by linking baseline information with the nationwide and continuously updated MS registry.^13^ The degree of misclassification of outcome is likely to be low and unrelated to fish consumption. Changes in fish consumption habits after baseline could be assessed by the follow-up questionnaire that was sent out in 2021. The response rate was lower in the EIMS follow-up study, however, there were no significant differences in baseline EDSS or at 5 years post-diagnosis among those who participated in the follow-up study and those who did not.

Information regarding fish consumption habits collected at baseline should be subjected to limited recall bias. It should be noted that information on fish preparation styles was not queried, which may influence the nutritional profile and health effects of fish consumption.

We were able to adjust for a large number of clinical and lifestyle variables. However, the inclusion of lifestyle variables did not significantly impact the association between fish consumption and MS disability progression, as each variable altered the estimated measure of association by less than 10%. To avoid over-adjusting our analysis, we only included age, sex, disease phenotype, disease duration, and treatment in our final model. Nonetheless, we provided a fully adjusted analysis in an online supplemental table 2. Despite these strengths, we cannot completely rule out residual confounding due to low granularity/possible misclassification or unknown confounders.

In conclusion, our findings suggest that higher fish consumption is associated with a reduced risk of disability progression in MS, likely due to the anti-inflammatory and neuroprotective properties of nutrients found in fish. These results highlight the potential importance of dietary habits in managing MS.

## Supplementary material

10.1136/jnnp-2024-335200 online supplemental file 1

## Data Availability

Data are available upon reasonable request.
