# Supplementary material for: Impact of fish consumption on disability progression in multiple sclerosis
Source: J Neurol Neurosurg Psychiatry. 2025 Feb 25;96(9):e335200. doi: 10.1136/jnnp-2024-335200 (PMC12418535; doi:10.1136/jnnp-2024-335200)
Supplement: online supplemental file 1 [file jnnp-96-9-s001.docx]

eTable 1. HR with 95% CI of having unfavorable outcomes post-diagnosis, by fish consumption habits *at diagnosis (n=2719)*.

| First clinical disease worsening (CDW) | | | | | |  |
| --- | --- | --- | --- | --- | --- | --- |
| Index score | N | Years (SD) | Outcome (%) | HR (95% CI)^1^ | HR (95% CI)^2^ | HR for trend (95% CI) |
| 2 | 332 | 6.3 (4.3) | 173 (52) | 1.0 (reference) | 1.0 (reference) | 0.94 (0.89-0.99) |
| 3 | 585 | 6.4 (4.4) | 292 (50) | 0.94 (0.78-1.14) | 0.89 (0.73-1.07) |  |
| 4 | 1142 | 6.5 (4.5) | 579 (51) | 0.95 (0.80-1.13) | 0.88 (0.74-1.04) |  |
| 5 | 436 | 6.6 (4.4) | 229 (53) | 0.94 (0.80-1.19) | 0.88 (0.72-1.08) |  |
| 6 | 224 | 6.9 (4.7) | 93 (42) | 0.75 (0.58-0.96) | 0.66 (0.51-0.86) |  |
| EDSS 3 | | | | | |  |
| Index score | N | Years (SD) | Outcome (%) | HR (95% CI)^1^ | HR (95% CI)^2^ | P for trend |
| 2 | 250 | 7.4 (4.5) | 92 (37) | 1.0 (reference) | 1.0 (reference) | 0.89 (0.83-0.96) |
| 3 | 462 | 7.6 (4.5) | 148 (32) | 0.82 (0.64-1.07) | 0.80 (0.62-1.04) |  |
| 4 | 875 | 7.8 (4.8) | 294 (34) | 0.85 (0.67-1.07) | 0.78 (0.62-1.00) |  |
| 5 | 335 | 8.4 (4.7) | 104 (31) | 0.77 (0.58-1.01) | 0.70 (0.53-0.94) |  |
| 6 | 170 | 8.1 (4.7) | 48 (28) | 0.71 (0.50-1.00) | 0.55 (0.39-0.79) |  |
| EDSS 4 | | | | | |  |
| Index score | N | Years (SD) | Outcome (%) | HR (95% CI)^1^ | HR (95% CI)^2^ | P for trend |
| 2 | 250 | 8.5 (4.4) | 38 (15) | 1.0 (reference) | 1.0 (reference) | 0.92 (0.83-1.01) |
| 3 | 462 | 8.8 (4.3) | 60 (13) | 0.83 (0.55-1.24) | 0.80 (0.50-1.12) |  |
| 4 | 875 | 8.8 (4.8) | 140 (16) | 0.99 (0.70-0.41) | 0.89 (0.63-1.29) |  |
| 5 | 335 | 9.1 (4.7) | 45 (13) | 0.84 (0.55-1.30) | 0.73 (0.47-1.13) |  |
| 6 | 170 | 9.1 (4.9) | 23 (14) | 0.80 (0.47-1.34) | 0.57 (0.33-0.96) |  |

^1^crude; ^2^adjusted for age at diagnosis, sex, residential area, ancestry, disease phenotype, disease duration, baseline EDSS, and disease-modifying therapy.

eTable 2. HR with 95% CI of having unfavorable outcomes post-diagnosis, by fish consumption habits *at diagnosis*, further adjusted for various lifestyle factors (n=2719).

| First clinical disease worsening (CDW) | | | | | |  |
| --- | --- | --- | --- | --- | --- | --- |
| Index score | N | Years (SD) | Outcome (%) | HR (95% CI)^1^ | HR (95% CI)^2^ | HR for trend (95% CI) |
| 2 | 332 | 6.3 (4.3) | 173 (52) | 1.0 (reference) | 1.0 (reference) | 0.95 (0.90-1.00) |
| 3 | 585 | 6.4 (4.4) | 292 (50) | 0.94 (0.78-1.14) | 0.90 (0.74-1.08) |  |
| 4 | 1142 | 6.5 (4.5) | 579 (51) | 0.95 (0.80-1.13) | 0.91 (0.77-1.08) |  |
| 5 | 436 | 6.6 (4.4) | 229 (53) | 0.94 (0.80-1.19) | 0.90 (0.78-1.17) |  |
| 6 | 224 | 6.9 (4.7) | 93 (42) | 0.75 (0.58-0.96) | 0.69 (0.55-0.91) |  |
| EDSS 3 | | | | | |  |
| Index score | N | Years (SD) | Outcome (%) | HR (95% CI)^1^ | HR (95% CI)^2^ | P for trend |
| 2 | 250 | 7.4 (4.5) | 92 (37) | 1.0 (reference) | 1.0 (reference) | 0.90 (0.84-0.97) |
| 3 | 462 | 7.6 (4.5) | 148 (32) | 0.82 (0.64-1.07) | 0.80 (0.62-1.04) |  |
| 4 | 875 | 7.8 (4.8) | 294 (34) | 0.85 (0.67-1.07) | 0.80 (0.64-1.04) |  |
| 5 | 335 | 8.4 (4.7) | 104 (31) | 0.77 (0.58-1.01) | 0.74 (0.58-1.04) |  |
| 6 | 170 | 8.1 (4.7) | 48 (28) | 0.71 (0.50-1.00) | 0.58 (0.42-0.86) |  |
| EDSS 4 | | | | | |  |
| Index score | N | Years (SD) | Outcome (%) | HR (95% CI)^1^ | HR (95% CI)^2^ | P for trend |
| 2 | 250 | 8.5 (4.4) | 38 (15) | 1.0 (reference) | 1.0 (reference) | 0.94 (0.84-1.03) |
| 3 | 462 | 8.8 (4.3) | 60 (13) | 0.83 (0.55-1.24) | 0.81 (0.51-1.15) |  |
| 4 | 875 | 8.8 (4.8) | 140 (16) | 0.99 (0.70-0.41) | 0.89 (0.65-1.35) |  |
| 5 | 335 | 9.1 (4.7) | 45 (13) | 0.84 (0.55-1.30) | 0.79 (0.52-1.26) |  |
| 6 | 170 | 9.1 (4.9) | 23 (14) | 0.80 (0.47-1.34) | 0.60 (0.36-1.05) |  |

^1^crude; ^2^adjusted for age at diagnosis, sex, residential area, ancestry, disease phenotype, disease duration, baseline EDSS, disease-modifying therapy, body mass index, physical activity, smoking, alcohol consumption, and sun exposure habits.

eTable 3. HR with 95% CI of having unfavorable outcomes post-diagnosis, by fish consumption habits among those with available vitamin D data *(n=1220)*.

| First clinical disease worsening (CDW) | | | | | |  |
| --- | --- | --- | --- | --- | --- | --- |
| Index score | N | Years (SD) | Outcome (%) | HR (95% CI)^1^ | HR (95% CI)^2^ | HR for trend (95% CI) |
| 2 | 150 | 7.2 (5.2) | 59 (61) | 1.0 (reference) | 1.0 (reference) | 0.95 (0.90-1.00) |
| 3 | 270 | 7.3 (5.3) | 115 (57) | 0.94 (0.78-1.13) | 0.89 (0.73-1.07) |  |
| 4 | 521 | 7.2 (5.4) | 208 (60) | 0.95 (0.80-1.12) | 0.90 (0.76-1.07) |  |
| 5 | 187 | 7.3 (5.1) | 73 (61) | 0.97 (0.80-1.19) | 0.92 (0.75-1.13) |  |
| 6 | 92 | 8.0 (5.9) | 40 (57) | 0.75 (0.58-0.97) | 0.68 (0.52-0.88) |  |

^1^crude; ^2^adjusted for age at diagnosis, sex, residential area, ancestry, disease phenotype, disease duration, baseline EDSS, disease-modifying therapy, vitamin D, and sampling month.

eTable 4. HR with 95% CI of having unfavorable outcomes post-diagnosis, by fish consumption habits at diagnosis among those without changes in fish consumption during *follow-up (n=1307)*.

| First clinical disease worsening (CDW) | | | | | |  |
| --- | --- | --- | --- | --- | --- | --- |
| Index score | N | Years (SD) | Outcome (%) | HR (95% CI)^1^ | HR (95% CI)^2^ | HR for trend (95% CI) |
| 2 | 131 | 6.1 (3.9) | 74 (56) | 1.0 (reference) | 1.0 (reference) | 0.91 (0.84-0.98) |
| 3 | 269 | 6.7 (4.6) | 137 (51) | 0.83 (0.62-1.10) | 0.76 (0.60-1.01) |  |
| 4 | 538 | 6.7 (4.4) | 266 (49) | 0.80 (0.62-1.04) | 0.72 (0.56-0.94) |  |
| 5 | 268 | 7.1 (4.6) | 147 (55) | 0.84 (0.65-1.12) | 0.75 (0.56-0.99) |  |
| 6 | 101 | 7.0 (5.0) | 39 (39) | 0.64 (0.43-0.94) | 0.55 (0.37-0.82) |  |
| EDSS 3 | | | | | |  |
| Index score | N | Years (SD) | Outcome (%) | HR (95% CI)^1^ | HR (95% CI)^2^ | P for trend |
| 2 | 105 | 7.3 (4.3) | 43 (41) | 1.0 (reference) | 1.0 (reference) | 0.87 (0.78-0.96) |
| 3 | 220 | 8.1 (4.6) | 69 (31) | 0.69 (0.47-1.02) | 0.63 (0.43-0.93) |  |
| 4 | 443 | 8.0 (4.7) | 154 (35) | 0.78 (0.56-1.09) | 0.65 (0.46-0.92) |  |
| 5 | 209 | 9.1 (4.9) | 68 (33) | 0.65 (0.45-1.00) | 0.58 (0.39-0.85) |  |
| 6 | 80 | 8.3 (5.1) | 22 (28) | 0.63 (0.38-1.05) | 0.46 (0.27-0.78) |  |
| EDSS 4 | | | | | |  |
| Index score | N | Years (SD) | Outcome (%) | HR (95% CI)^1^ | HR (95% CI)^2^ | P for trend |
| 2 | 105 | 8.4 (4.1) | 18 (17) | 1.0 (reference) | 1.0 (reference) | 0.87 (0.74-1.02) |
| 3 | 220 | 9.2 (4.5) | 26 (12) | 0.63 (0.35-1.15) | 0.56 (0.31-1.03) |  |
| 4 | 443 | 9.1 (4.6) | 71 (16) | 0.89 (0.53-1.49) | 0.72 (0.43-1.20) |  |
| 5 | 209 | 9.8 (4.8) | 25 (12) | 0.61 (0.33-1.12) | 0.51 (0.27-0.94) |  |
| 6 | 80 | 9.5 (5.2) | 10 (13) | 0.70 (0.32-1.53) | 0.47 (0.21-1.04) |  |

^1^crude; ^2^adjusted for age at diagnosis, sex, residential area, ancestry, disease phenotype, disease duration, baseline EDSS, and disease-modifying therapy.

eTable 5. Baseline characteristics of the overall sample, follow-up responders, and non-responders.

|  | Baseline questionnaire | Responders to follow-up questionnaire | Non-responders to follow-up questionnaire | P value^1^ |
| --- | --- | --- | --- | --- |
| N | 2719 | 1917 | 1000 |  |
| Age at diagnosis (SD) | 38 (11) | 38 (11) | 37 (12) | 0.07 |
| Female, n (%) | 1950 (72) | 1232 (72) | 487 (72) | 0.94 |
| Nordic origin, n (%) | 2173 (81) | 1419 (83) | 754 (78) | 0.008 |
| Treatment (%) | 2573 (95) | 1639 (95) | 934 (93) | 0.03 |
| MS phenotype Relapsing, n (%) Progressive, n (%) Unknown, n (%) | 2542 (93) 131 (4.8) 46 (1.7) | 1632 (95) 73 (4.3) 14 (0.8) | 910 (91) 58 (5.8) 32 (3.2) | 0.45 |
| Disease duration, years (SD) | 2.6 (3.8) | 2.8 (3.9) | 2.4 (3.4) | 0.26 |
| Baseline EDSS (SD) | 1.8 (1.4) | 1.8 (1.4) | 1.8 (1.5) | 0.17 |
| Mean fish consumption frequency score (SD) | 3.9 (1.1) | 3.9 (1.1) | 3.8 (1.1) | 0.12 |
| Sun exposure index (SD) | 6.2 (1.8) | 6.2 (1.8) | 6.2 (1.9) | 0.85 |
| Vitamin D, ng/mL (SD)^1^ | 62 (27) | 63 (27) | 59 (26) | 0.05 |
| Past IM, n (SD)^2^ | 485 (18) | 310 (18) | 175 (18) | 0.46 |
| Physical activity (SD) | 2.3 (1.0) | 2.4 (0.9) | 2.2 (1.0) | <0.0001 |
| Body mass index (SD) | 25.1 (4.8) | 25.1 (4.7) | 25.1 (5.1) | 0.43 |
| Current smoking, n (%) | 614 (23) | 290 (19) | 324 (29) | <0.0001 |
| Alcohol (gram/week, SD) | 43 (63) | 44 (63) | 41 (64) | 0.01 |

^1^p value for difference between respondents and non-respondents
